# Genome-Wide Analysis of the Zn(II)_2_Cys_6_ Zinc Cluster-Encoding Gene Family in *Tolypocladium* *guangdongense* and Its Light-Induced Expression

**DOI:** 10.3390/genes10030179

**Published:** 2019-02-26

**Authors:** Chenghua Zhang, Hong Huang, Wangqiu Deng, Taihui Li

**Affiliations:** 1 State Key Laboratory of Applied Microbiology Southern China, Guangdong Provincial Key Laboratory of Microbial Culture Collection and Application, Guangdong Open Laboratory of Applied Microbiology, Guangdong Institute of Microbiology, Guangzhou 510070, China; zhangch@gdim.cn (C.Z.); huanghong@gdim.cn (H.H.); 2 College of Life Science, University of Chinese Academy of Sciences, Beijing 100190, China

**Keywords:** C6 zinc gene, *Cordyceps*, fruiting body development, expression pattern, metabolic process

## Abstract

The Zn(II)_2_Cys_6_ zinc cluster gene family is a subclass of zinc-finger proteins, which are transcriptional regulators involved in a wide variety of biological processes in fungi. We performed genome-wide identification and characterization of Zn(II)_2_Cys_6_ zinc-cluster gene (C6 zinc gene) family in *Tolypocladium* *guangdongense*, *Cordyceps* *militaris* and *Ophiocordyceps* *sinensis*. Based on the structures of the C6 zinc domains, these proteins were observed to be evolutionarily conserved in ascomycete fungi. We focused on *T.* *guangdongense*, a medicinal fungus, and identified 139 C6 zinc genes which could be divided into three groups. Among them, 49.6% belonged to the fungal specific transcriptional factors, and 16% had a DUF3468 domain. Homologous and phylogenetic analysis indicated that 29 C6 zinc genes were possibly involved in the metabolic process, while five C6 zinc genes were supposed to be involved in asexual or sexual development. Gene expression analysis revealed that 54 C6 zinc genes were differentially expressed under light, including two genes that possibly influenced the development, and seven genes that possibly influenced the metabolic processes. This indicated that light may affect the development and metabolic processes, at least partially, through the regulation of C6 zinc genes in *T.* *guangdongense*. Our results provide comprehensive data for further analyzing the functions of the C6 zinc genes.

## 1. Introduction

Zinc cluster proteins form one of the largest families of transcriptional regulators in eukaryotes, performing a wide variety of functions in transcriptional and translational processes. Based on the zinc finger binding motifs, zinc cluster proteins can be categorized into three main classes, Cys_2_His_2_ (C2H2), Cys_4_ (C4), and Cys_6_ (C6) [1]. Among them, the C6 type of zinc cluster proteins has attracted wide attention. These proteins contain a DNA-binding domain (DBD) which possesses the well-conserved CysX_2_CysX_6_CysX_5-12_CysX_2_CysX_6-8_Cys motif with cysteines binding to two zinc atoms, and therefore they are also called Zn(II)_2_Cys_6_ or C6 zinc proteins [1,2]. 

Previous studies showed that C6 zinc proteins act as global regulators both on primary and secondary metabolism. These proteins are best studied in the budding yeast *Saccharomyces cerevisiae*, as well as in some model fungi, such as *Aspergillus nidulans, Neurospora crassa*, and other *Aspergillus* spp. They are shown to be involve in carbon utilization [3,4], gluconeogenesis [5,6], respiration [7,8], amino acid metabolism [1], vitamin synthesis [9], nitrogen utilization [10], ergosterol biosynthesis and uptake [11,12], as well as the stress response [13]. Besides, some C6 proteins are also involved in secondary metabolite production as transcription activators to regulate the expression of clustering genes [4]. In addition to the roles mentioned above, some C6 zinc proteins have been characterized to be involved in asexual and sexual development. In *A. nidulans*, C6 zinc gene *OEFC* positively regulated the asexual development, the absence of it led to producing undifferentiated aerial hyphae, and failed to develop conidiophores [14]. Another C6 zinc encoding gene *SFGA* negatively regulated the conidia production, as its overexpression inhibited conidiation [15]. In the sexual stage, overexpression of *ROSA* resulted in colonies with fluffy, cotton-like hyphae [16], while deletion of *NOSA* blocked the primordial stage, and occasionally produced minute cleistothecia containing fertile ascospores [17]. Besides, in the homothallic ascomycete *Sordaria macrospora*, deletion of *PRO1* prevented the transition of primordia into mature fruiting bodies [18], and a similar function of its homologue was also detected in *Podospora anserina* [19]. Interestingly, all the above mentioned C6 zinc proteins contain a DUF3468 domain (Pfam: PF11951), which is present in a family of putative fungal transcription factors [4], implying that C6 zinc proteins with DUF3468 domain are crucial in regulating the fungal development, thus need to be further investigated. 

*Tolypocladium* is a genus in Ophiocordycipitaceae (Hypocreales, Sordariomycetes), which is a large family of fungi that were considered to have potential applications in medicine and agriculture [20,21,22]. In this genus, a few species are the primarily pathogens of insects, while most species are parasites of truffle fruiting bodies [20,22]. *T. guangdongense* (synonym *Cordyceps guangdongensis,* abbreviated to CCG), which belong to the genus Tolypocladium, is a typical medicinal fungus. The importance and potential application of this fungus is not only owing to the fact that it has been authenticated as novel food by the State Food and Drug Administration of the Ministry of Public Health of China, but also to its various biological activities of fruiting bodies, such as antiviral activity against influenza virus H9N2 infections [23], longevity-increasing activity [24], curative effect on chronic renal failure [25], anti-fatigue effect [26], and anti-inflammatory effect [27]. Although the majority of active ingredients in *T. guangdongense* are still unknown, some active ingredients, such as cordycepic acids, adenosine and polysaccharides have been identified in this fungus [28]. As a medicinal fungus, the fruiting body of *T. guangdongense* is the major form or raw material for industrial production and commercialization. Besides, the significant medicinal activities of its fruiting body attributed to the various known and unknown active ingredients. However, the fruiting body has a complex developmental process, and little is known about the regulation mechanism of the active components. 

Now, with the development of omics technology, more numerous genomes of fungi within the genus Tolypocladium have been available, including *Tolypocladium ophioglossoides* [29] and *Tolypocladium inflatum* [21]. These genomic resources provided a new insight into better understanding of the relevance of phenotypic characters and genetic mechanisms. The genome sequence of *T. guangdongense* was also reported [30]. In an effort to understand the possible regulatory mechanisms of fruiting body development and active component metabolism, we identified C6 zinc cluster genes at the genome level in *T. guangdongense* and two other allied species *C. militaris* and *O. sinensis*, and compared their number, type, and conserved domain sequences. Furthermore, this research focused on C6 zinc cluster genes in *T. guangdongense*, analyzing the sequence features, chromosomal locations, phylogenetic relationships, possible functions, and expression pattern under light condition. These results may provide useful information for further functional investigations of the C6 zinc cluster gene family.

## 2. Materials and Methods 

### 2.1. Identification of C6 Zinc Genes and C6-TFs

To identify the Zn(II)_2_Cys_6_ zinc cluster genes, ScanProsite, a web-based tool for detecting PROSITE signature matches in protein sequences (http://www.expasy.org/tools/scanprosite/), was used to predict the conserved Zn(II)_2_Cys_6_ domain (PS00463 and PS50048) proteins based on the genome database of *T. guangdongense*, previously known as *Cordyceps guangdongensis* [30] (abbreviated to CCG) according to the method described by De Castro et al [31]. Each candidate sequence of C6 zinc proteins was further confirmed by domain analysis using the Pfam protein family database (http://pfam.xfam.org) [32] and SMART databases (http://smart.emblheidelberg.de/) [33]. C6 zinc proteins which contained the fungal specific transcription factor domain (C6-TFs) were also analyzed by Pfam and SMART databases. Identification of C6-proteins and C6-TFs in *C. militaris* and *O. sinensis* were performed similarly based on the genome sequences from the Ensembl fungi database (http://fungi.ensembl.org/index.html). 

### 2.2. Sequence Analysis of C6 Zinc Proteins

C6 zinc proteins were classified based on the number of amino acid residues in the variable subregions of the well-known C6 zinc cluster domain. The sequences of amino acids and CDS lengths were obtained from *T. guangdongense* genome. Exon/intron structures were obtained by comparing the coding sequence and the corresponding genomic DNA sequence of C6 zinc genes. Isoelectric point (pI) and molecular weight (Mw) of C6 zinc proteins were calculated using the ExPASy tool (http://web.expasy.org/compute pi/). The subcellular localization of each protein was analyzed with BaCelLo Prediction (http://gpcr.biocomp.unibo.it/bacello/pred.htm).

### 2.3. Chromosomal Mapping and Protein Motif Analysis of C6 Zinc Genes

The physical location data of the Zn(II)_2_Cys_6_ zinc cluster-encoding genes on the chromosome were retrieved from the *T. guangdongense* genome database. MapInspect software (http://www.plantbreeding.wur.nl/uk/sofware-mapinspect.html) was used to generate chromosomal distribution images for these C6-TFs in *T. guangdongense* [34]. The conserved motifs of the C6-TFs were investigated using the online MEME program (http://meme-suite.org/). The analysis was performed with a set of parameters as follows: Max motif width was set to 50 and the maximum number of motifs was set to 10. Only the motifs with *p*-values < 10^−6^ and no overlap with each other were reported. 

### 2.4. Sequence Alignment and Phylogenetic Tree Construction

Multiple alignments of C6 zinc protein sequences were performed using the ClustalW program [35]. Phylogenetic trees were constructed based on the neighbor-joining (NJ) method with a Kimura2-parameter model using MEGA 5.0 [36]. The stability of the internal nodes was assessed with a bootstrap analysis of 1000 replicates. The phylogenetic tree was visualized using iTOL (http://itol.embl.de/help.cgi). For the functional analysis, homologous proteins were obtained by a BLASTP search run against the GenBank database. 

### 2.5. Expression Analysis of C6 Zinc Cluster Genes under Different Light Conditions

To analyze the expression patterns of C6 genes, transcriptome analysis of C6 genes under different light conditions was conducted. The strain of *T. guangdongense* (GDIM_C05423) was cultured on PDA medium at 23 ± 1 °C for four weeks. The mycelia were transferred to a new PDA medium with cellophane overlays and was incubated at 23 ± 1 °C under continuous dark conditions for another four weeks, then incubated at 23 ± 1 °C for another 24 h with different light conditions (dark, light treatment for 0.5 h, 4 h, 6 h). Samples were collected at the indicated time points for RNA-seq analysis. Total RNA was extracted using the Trizol kit (Promega, Medison, WI, USA) following the manufacturer’s instructions. The RNA quality was verified using 2100 Bio-analyzer (Agilent Technologies, Santa Clara, CA, USA) and also checked by RNase-free agarose gel electrophoresis. The cDNA library was sequenced on the Illumina sequencing platform (Illumina HiSeq 2000, San Diego, CA, USA) using paired-end technology by Gene Denovo. Gene expression of the C6 zinc genes were selected from the RNA-seq profiles, and the differentially expressed genes were estimated by fold-change. Transcripts with fold change in expression ≥ 2.0 and with a *p*-value < 0.05 were considered significant. Hierarchical clustering and heat map drawing were performed via the OmicShare platform with the FPKM (fragments per kilobase of transcript per million mapped reads) values.

## 3. Results

### 3.1. Identification of C6 Zinc Cluster Genes in *Tolypocladium guangdongense*

**Figure 1:**
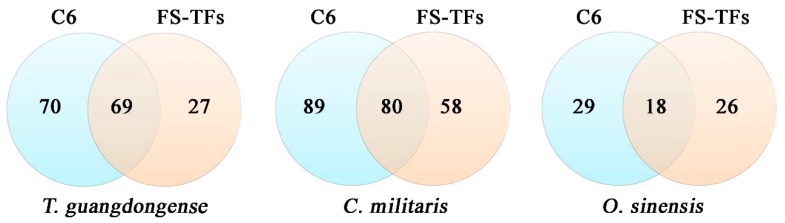
Estimates of C6 zinc genes and fungal specific transcription factors (FS-TFs) in *Tolypocladium guangdongense*, *Cordyceps militaris* and *Ophiocordyceps sinensis* genomes by automatic and manual analyses. The detail information was shown in Table S2.

The whole genome sequence of *T. guangdongense* reported previously, and two other *Cordyceps* (*C. militaris* and *O. sinensis*) genome sequences from ensembl fungi database were used for a genome-wide search of Zn(2)-C6 fungal-type DNA-binding domain encoding genes. After further scrutinizing by domain analysis using Pfam protein family database and SMART databases in order to remove the unrelated, as well as mis-predicted sequences, 139 C6 zinc cluster-encoding genes were identified in *T. guangdongense*, accounting for 1.51% of the total predicted proteins (Figure 1). Similar searches were performed against the genome databases of *C. militaris* and *O. sinensis*, 169 and 47 C6 zinc cluster-encoding genes were found, which were 1.75% and 0.67% of each total predicted protein, respectively (Table S1). Among the C6 zinc genes in *T. guangdongense*, 49% of genes were belonged to the fungal specific transcription factors (FS-TFs), accounting for about 71% of the total fungal specific transcription factors. While in *C. militaris* and *O. sinensis*, 47% and 38% genes were fungal specific transcription factors, accounting for about 60% and 41% of the total fungal specific transcription factors, respectively (Table S2). In consideration of the genome size of these species, the average distributions of C6 zinc genes in the genome region or across chromosome were variable. Assuming the C6 zinc genes distributed evenly, one C6 zinc gene would reside in each 208-kb genomic region of *T. guangdongense*. The distribution density of C6 zinc genes in *T. guangdongense* was higher than that in *C. militaris* (190-kb). Considering the incomplete genome sequence, the total number of C6 zinc genes predicted in *O. sinensis* has been likely underestimated. 

### 3.2. Sub-Grouping of C6 Zinc Proteins of *Tolypocladium guangdongense*

According to previous reports, the numbers of amino acid residues in the well-known Gal4 zinc cluster domain between C1-C2, C2-C3 and C4-C5 are always 2, 6, and 2, respectively. While the loop regions between C3-C4 and C5–C6 show variability in length and sequences. Based on the variable subregions, the predicted C6 zinc proteins could be classified into several subgroups with Cys-X_2_-Cys-X_6_-Cys-X_5-12_-Cys-X_2_-Cys-X_6-12_-Cys pattern (Table S3). Proteins with C-2-C-6-C-6-C-2-C-6-C pattern are most abundant followed by those with C-2-C-6-C-5-C-2-C-6-C pattern, which takes on a common phenomenon among the three species (Table 1). The ratio of predicted proteins with the mentioned patterns in *T. guangdongense* is about 46.8% and 18.7% of the all C6 zinc proteins, respectively. Other less common patterns, include C-2-C-6-C-5-C-2-C-8-C, C-2-C-6-C-8-C-2-C-6-C, C-2-C-6-C-9-C-2-C-6-C, and C-2-C-6-C-7-C-2-C-6-C, accounted for 10.8%, 7.9%, 4.3% and 3.6% of the all C6 zinc proteins, respectively. Comparison of the three species, three unique patterns C-2-C-6-C-17-C-2-C-6-C, C-2-C-6-C-17-C-2-C-6-C and C-2-C-6-C-5-C-2-C-5-C were found in *T. guangdongense*, while five unique patterns C-2-C-6-C-11-C-2-C-7-C, C-2-C-6-C-12-C-2-C-6-C, C-2-C-6-C-6-C-2-C-7-C, C-2-C-6-C-6-C-2-C-11-C and C-2-C-6-C-6-C-2-C-9-C were found in *C. militaris*. In addition, *C. militaris* possessed the most abundant C6-type sequences of variable subregions with at least 21 patterns, followed by *T. guangdongense* with 18 patterns, and *O. sinensis* with 10 patterns.

### 3.3. Characteristics and Classification of C6 Zinc Proteins in *Tolypocladium guangdongense*

Detailed information of the 139 C6 zinc genes is shown in Table S4, including numbers of exons, encoding proteins sizes, physicochemical parameters and subcellular localization. The predicted C6 zinc genes in *T. guangdongense* encode proteins ranging from 104 to 1395 amino acids in length, with an average size of 724. The predicted molecular weights of the C6 zinc proteins were ranged from 10.8 to 153.4 kDa (average 79.5 kDa) and the predicted protein isoelectric points (pI) are below 11. As shown in Table S4, based on the analysis of the predicted subcellular localization, the C6 zinc proteins could be categorized into four groups. Most of the C6 zinc proteins were located at the nucleus, accounting for 62.6% of the total C6 zinc proteins, followed by those which were predicted to be localized to the cytoplasm (23%), mitochondrion (9.4%), and secretory (5.0%).

**Figure 2:**
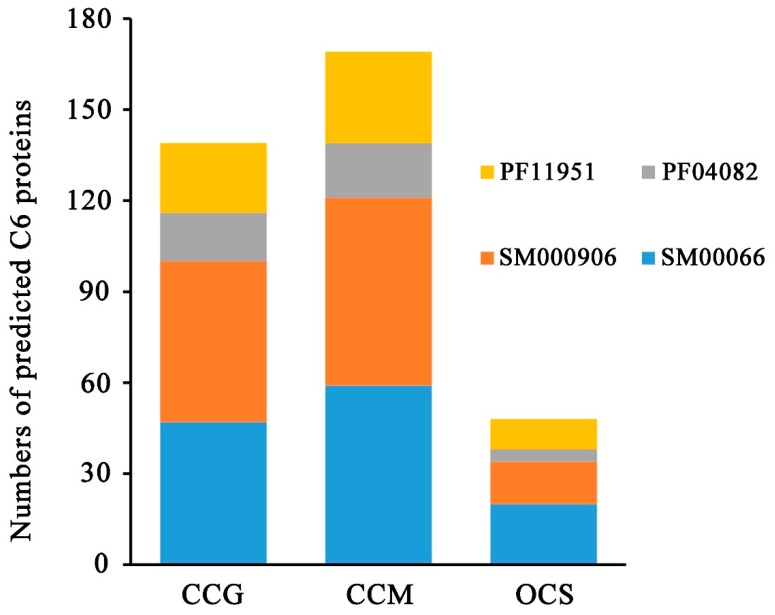
Classification of the identified C6 zinc proteins in *T. guangdongense* (CCG), *C. militaris* (CCM) and *O. sinensis* (OCS).

Among the 139 C6 zinc proteins in *T. guangdongense*, 69 proteins were verified as C6-TFs using the Pfam and SMART databases (Table S3), both of which contained the Gal4 and fungal specific TF domain (C6-FSP-TFs). The C6-FSP-TFs accounted for 49.6% of the total C6 zinc proteins in *T. guangdongense*, while the proportion of C6-FSP-TFs in *C. militaris* was slight lesser (46.5%) (Table S2). The proportion of C6-FSP-TFs in C6 zinc proteins was the lowest (37.5%) in *O. sinensis*, compared to the other two species. Based on the fungal specific transcription factor domains, the C6-FSP-TFs could be classified into two subgroups (Figure 2). Group Ⅰ contains an SM000906 type transcription factor domain, and group Ⅱ contains a PF04082 type transcription factor domain. By comparison, *T. guangdongense* and *C. militaris* possessed a similar proportion of C6-FSP-TFs, with 49.6% and 47.3% of the total C6 zinc proteins. C6-FSP-TFs in *O. sinensis* accounted for 38.3% of the total C6 zinc proteins. Among the three species, the overwhelming majority of the C6-FSP-TFs is the Group Ⅰ. Searching for the conserved domain, 70 C6-encoding proteins did not have the fungal specific TF domain in *T. guangdongense* (Figure 2). Of which, 23 were found to encode a unique domain called DUF3468 (DUF, domain of unknown function), also the Pfam domain with an accession number PF11951. Except for the three genes, all DUF3468 proteins had the C6 pattern of C-2-C-6-C-6-C-2-C-6-C. The predicted protein encoded by CCG_02279 has two C6 zinc domains, with the C6 pattern of C-2-C-6-C-5-C-2-C-6-C and C-2-C-6-C-6-C-2-C-6-C. The exceptive three (CCG_00047, CCG_01361, and CCG_06067) have the C6 pattern of C-2-C-6-C-9-C-2-C-6-C, C-2-C-6-C-17-C-2-C-6-C and C-2-C-6-C-5-C-2-C-6-C, respectively (Table S3). In *C. militaris*, 89 C6-encoding proteins did not have the fungal specific TF domain, of which, 30 C6-encoding genes have the DUF3468 domain. Except for two genes, all DUF3468 proteins had the C6 pattern of C-2-C-6-C-6-C-2-C-6-C. The predicted protein encoded by CCM_00582 has two C6 zinc domains, with the same C6 pattern as CCG_02279. The exceptions (CCM_02104 and CCM_01975) have the C6 pattern of C-2-C-6-C-6-C-2-C-11-C and C-2-C-6-C-8-C-2-C-6-C, respectively (Table S3). DUF3468 domain proteins in *O. sinensis* accounted for a relatively large proportion of 42.6%, and except one (OCS_05587) has the C6 pattern of C-2-C-6-C-5-C-2-C-8-C, all DUF3468 proteins had the C6 pattern of C-2-C-6-C-6-C-2-C-6-C. These results showed that the C6 patterns of DUF3468 proteins are highly conserved in three species.

### 3.4. Chromosomal Distribution of C6 Zinc Genes in *Tolypocladium guangdongense* Genome 

**Figure 3:**
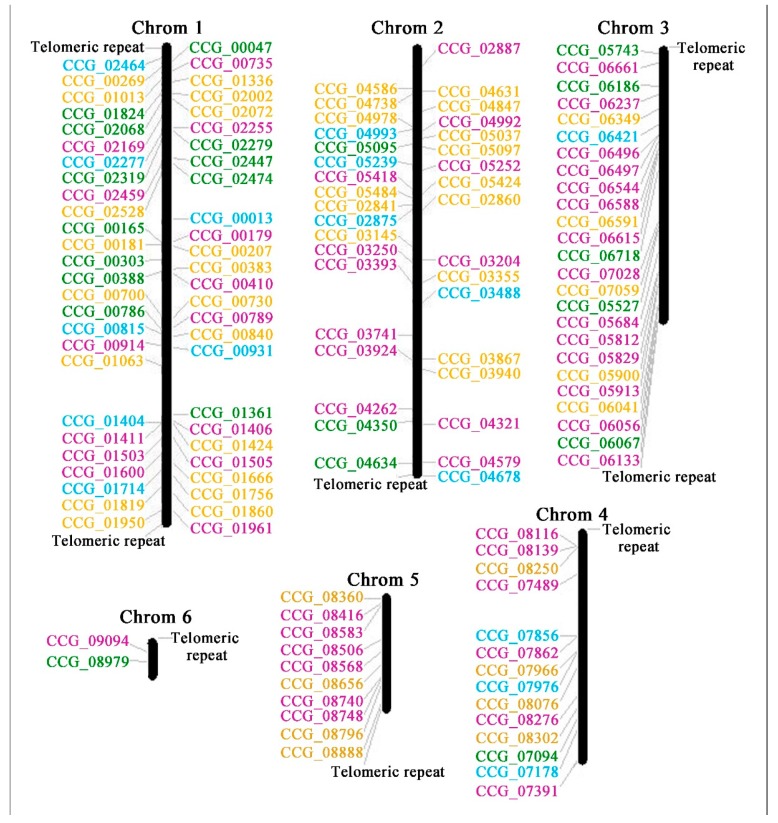
Genomic distribution of C6 zinc domain genes in *T. guangdongense*. 139 genes are located on six chromosomes. The numbers at the right side of the chromosomal bar indicate the names of C6 zinc domain genes and the corresponding position on the chromosome (megabase pairs; Mb) are given in Table S4. The “SM000906” type genes are represented in pink, “PF04082” type genes are in blue, “PF11951” type genes are in green and other “SM00066” type genes are in yellow color.

In order to examine the genome distribution of C6 zinc genes, chromosomal mapping was performed. As shown in Figure 3, the identified C6 zinc genes were located on six chromosomes indicating a diverse distribution. Chromosome 1 had the largest number of C6 zinc domain genes with 52 members, followed by chromosome 2 and 3, with 35 and 25 members respectively. Fifteen C6 zinc genes were located on chromosome 4, and ten members were located on chromosome 5. Only two C6 zinc genes were located on chromosome 6. Besides, in chromosome 1, about 38% C6 zinc genes belonged to the fungal specific transcription factors (including 13 SM000906 domain FS-TFs and 7 PF04082 domain FS-TFs), 25% C6 zinc genes contained the DUF3468 domain, while the rest were only contained the Gal4 domain (SM00066). The distribution of C6 zinc genes on chromosome 2, 3 and 4, showed similar patterns with the most number of FS-TF genes, followed by SM00066 type genes and DUF3468 type genes. On chromosome 4, there was one DUF3468 type gene detected, while no DUF3468 type genes were located on chromosome 5. Only one SM00066 type gene and one DUF3468 type gene were located on chromosome 6.

### 3.5. Functional Analysis of C6 Zinc Proteins Associated with Metabolic Process in *Tolypocladium guangdongense*

According to the BLAST analysis, 29 C6 zinc proteins were predicted to be associated with metabolic processes (Table 2 and Table S5). Majority of them were involved in regulating primary metabolic processes, while a few of them were predicted to be involved in secondary metabolite biosynthesis pathways. Homology analysis showed that six genes were predicted to be involved in carbon utilization, including xylan, cellulose, sucrose, maltose, and mannose utilization. CCG_08139 was the homologous gene of *XLNR* in *A. niger* or *A. oryzae* that controled the expression of xylanolytic enzymes coding genes. CCG_06056, CCG_06421, and CCG_02169 were the homologous genes of *SUC1* in *Candida albicans*, regulating genes involved in sucrose metabolism. There were two genes possibly associated with degradation of the cell wall components; CCG_08276 encoded the protein of chitinase 1 precursor, whereas CCG_05812 was homologous to *CTF1β* regulating the expression of cutinase genes as activators. Another two genes (CCG_02875 and CCG_02255) were possibly responsible for nitrogen utilization, with their homologous genes (*OTam*/*TamA*, *DAL81*) being well-characterized in *S. cerevisiae* and *A. nidulans*. Three genes were likely to participate in ergosterol biosynthesis or uptake, two of them were homologous to *UPC2* in *S. cerevisiae*, and one was homologous to *ECM22* in *S. cerevisiae*. Four genes were predicted to be involved in acetate utilization, with one gene was high homologous to *FACβ*, and the rest highly homologous to *acu-15* in *N. crassa*. Furthermore, two genes (CCG_07862 and CCG_07856) were located at the Indole-T1pks type gene cluster, as neighboring genes of the backbone gene CCG_07857 (encoding cytochrome P450 enzymes) and the transport-related gene CCG_07863 (encoding MFS-domain transporter), respectively. Homology analysis indicated that the amino acid sequence of CCG_07856 had 50% identity with *Penicillium aethiopicum vrtR2*, and amino acid sequence of CCG_07862 had 66% identity with *P. aethiopicum VRTR1*. Besides, some genes also participated in other metabolic pathways, including amino acid metabolism, sulfate assimilation, sulfonate metabolism, purine utilization, pyrimidine utilization, and quinic acid utilization.

### 3.6. Function Analysis of C6 Zinc Proteins Associated with Fruiting Body Development in *Tolypocladium guangdongense*

**Figure 4:**
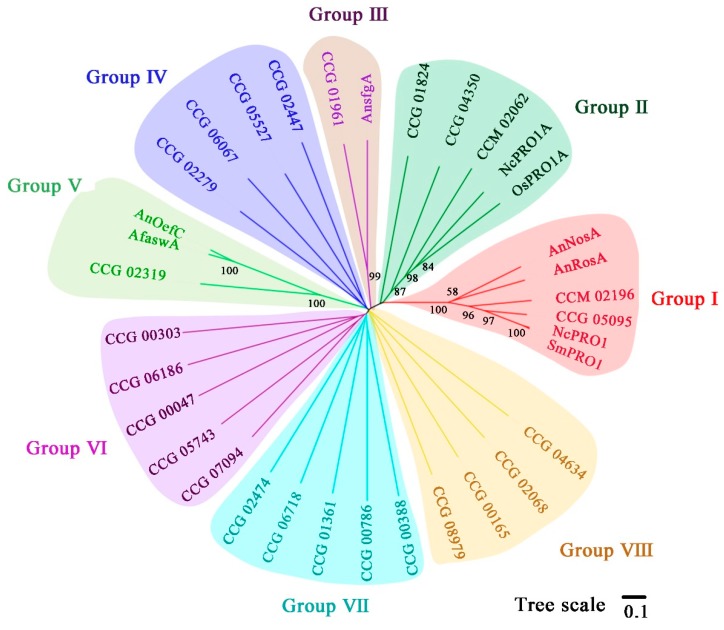
Phylogenetic relationship of 34 DUF3468 domain contained C6 zinc proteins in *T. guangdongense.* Amino acid sequences were aligned using ClustalW, and phylogenetic tree were generated by the neighbor-joining (NJ) method using MEGA 5.0. Numbers at the branch point of the node represent the value resulting from 1000 replications. All positions with less than 50% site coverage were eliminated. CCG, *T. guangdongense*; CCM, *C. militaris*; Sm, *S. macrospora*; Nc, *N. crassa*; An, *A. nidulans*; Os, *O. sinensis*; Af, *Aspergillus flavus*. GenBank numbers of other proteins are listed as follows: OsPRO1A, EQL03797; NcPRO1A, CAC86433; AnRosA, CAD58393; CCM_02062, XP_006667279; AnsfgA, AAY99779; SmPRO1, CAB52588; NCPRO1, AJ238440; AnNosA, CAJ76908; AfaswA, XP_002373431; CCM_02196, XP_006667411.

Based on the central roles of DUF3468 type C6 zinc proteins in asexual or sexual development of filamentous fungi, phylogenetic tree was constructed using full-length amino acid sequences. In total, 23 sequences from *T. guangdongense*, four sequences from *A. nidulans*, two sequences from *C. militaris*, two sequences from *N. crassa*, and one sequence each from *A. flavus*, *S. macrospora*, and *O. sinensis*, were assessed in the phylogenetic tree (Figure 4). The 34 DUF3468 domain containing C6 zinc proteins were classified into six groups. Group Ⅰ contained six proteins, which were the orthologs of *PRO1* in *S. macrospora* involved in fungal sexual development. The amino acid sequence of CCG_05095 showed 46%, 68%, 68% and 70% identities with *NosA* in *A. nidulans*, CCM_02196 in *C. militaris* (*CmPRO1*), *PRO1* in *S. macrospora*, and *PRO1* in *N. crassa*, respectively, all of these proteins contained the nuclear localization signal (NLS) sequences IKNIIKRKKL (Figure S1). The amino acid sequence of CCG_04350 and those of the *PRO1A* in *N. crassa* and *O. sinensis* formed one cluster with 87% approval rating (group Ⅱ). The amino acid sequence of CCG_01824 showed less than 50% approval rating with those of *PRO1* orthologs in group Ⅰ, but it shared about 45% identity with *PRO1A* in *Fusarium*. The amino acid sequence of CCG_01961 was clustered into an independent branch with *sfgA* in *A. nidulans* forming the group Ⅲ. The group Ⅴ contained three members, and the amino acid sequence of CCG_02319 shared 100% bootstrap support value with the *OEFC* orthologs in *A. nidulans* and *A. flavus*, and exhibited higher than 65% identities with other *OEFC* orthologs in *O. sinensis*, *Metarhizium robertsii*, and *Purpureocillium lilacinum*. The rest DUF3468 domain proteins with unknown function were roughly classified into four groups (Groups IV, VI and VII), and further work is needed to explore their functions.

**Figure 5:**
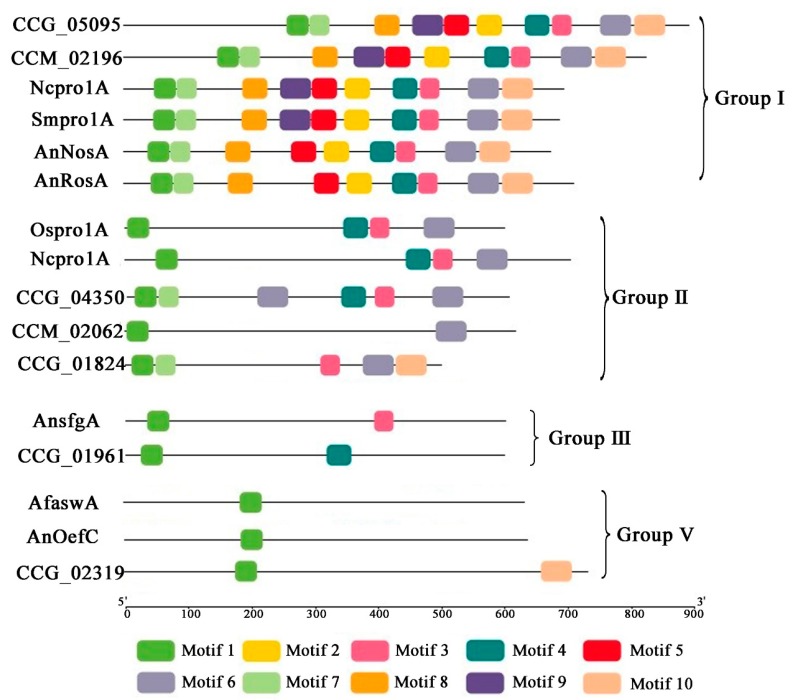
Conserved motifs in the DUF3468 domain contained C6 zinc proteins. Ten conserved motifs in all proteins were identified through the multiple EM for motif elicitation (MEME) analysis and were indicated in different colored boxes. CCG, *T. guangdongense*; CCM, *C. militaris*; Sm, *S. macrospora*; Nc, *N. crassa*; An, *A. nidulans*; Os, *O. sinensis*; Af, *Aspergillus flavus*. GenBank numbers of the related proteins are the same as Figure 4. Scale bar indicates a number of amino acids (aa). For details of motifs refer to Table S6 and Figure S2.

To further ascertain the relationship between C6 zinc proteins in *T. guangdongense* and the known functional C6 zinc proteins in other fungi, motif scan analysis were conducted with DUF3468 domain containing C6 zinc proteins (Figure 5 and Figure S2). The results showed that PRO1 protein from *T. guangdongense* (TgPRO1) had similar functional motifs consisting of highly conserved regions with NcPRO1, SmPRO1, CmPRO1, AnNosA and AnRosA, including one Zn_clus motif (motif 1) and six different Fungal_trans_2 motifs (motif 2–6, 10). These results implied that TgPRO1 may have a similar function as PRO1 from other fungi. PRO1A protein from *T. guangdongense* (TgPRO1A) shared similar functional motifs consisting of highly conserved regions with NcPRO1and OsPRO1, including one Zn_clus motif (Motif 1) and three different Fungal_trans_2 motifs (Motif 3, 4, 6). Besides, TgPRO1A contained two Fungal_trans_2 motifs of the same type (Motif 6). Except for the Zn_clus motif, TgsfgA (CCG_01961) contained a different type of Fungal_trans_2 Motif compared to AnsfgA, whereas the TgOefC (CCG_01961) also possessed another Fungal_trans_2 motif (motif 10). These results suggested that the TgsfgA and TgOefC possibly have more other functions. 

### 3.7. Expression Profile of C6 Zinc Proteins in *Tolypocladium guangdongense* under Light Conditions

**Figure 6:**
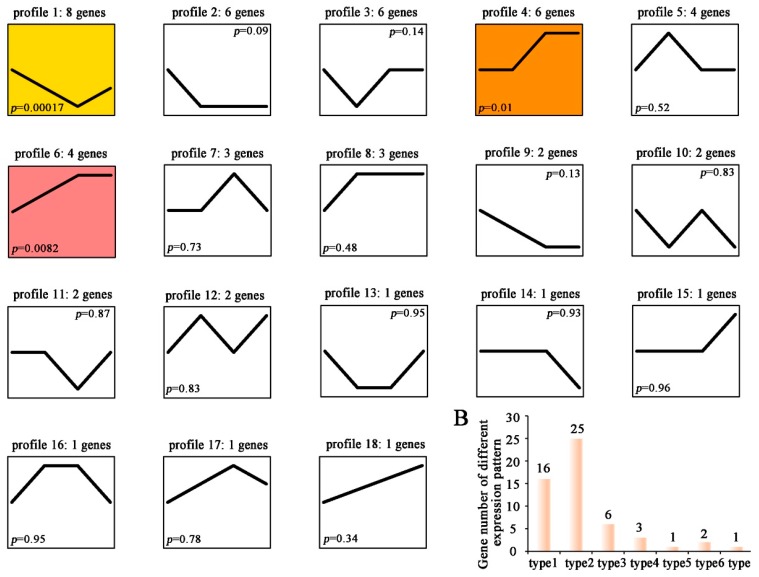
Expression profiles of C6 zinc domain genes under different light conditions in *T. guangdongense*. (**A**), Expression patterns of differentially expressed genes; (**B**), Gene classification based on the Expression patterns. Type 1 represented genes immediately increased expression levels after light treatment. Type 2 represented genes immediately decreased expression levels after light treatment. Type 3 represented genes showing no change in expression levels when light treatment for 30 min, but increased firstly and then stabilized expression pattern after light treatment for 30 min. Type 4 represented genes showing no change in expression levels when light treatment for 30 min, but increased firstly and then returned to the starting level after light treatment for 30 min. Type 5 represented gene showing no change in expression levels at first, but up-regulation after light treatment for four hours. Type 6 represented genes showing no change in expression levels when light treatment for 30 min, but decreased firstly and then returned to the starting level after light treatment for 30 min. Type 7 represented gene showing no change in expression levels at first, but down-regulation after light treatment for four hours.

In order to gather more information about the potential role of C6 zinc proteins in *T. guangdongense*, we analyzed the expression of C6 zinc genes under light conditions by transcriptome sequencing (Table S7). Expression trend analysis based on the Log2 value indicated that 54 genes were differentially expressed after light treatment, and could be divided into 18 profiles (Figure 6A). Among the 18 profiles, the expression pattern of three profiles (1, 4, and 6) showed a significant difference with the P value less than 0.05. Based on the expression pattern, all differentially expressed genes could be categorized into seven types (Figure 6B). Type one contained 16 members with immediately increased expression levels after light treatment, while type two contained 25 members with immediately decreased expression levels after light treatment. Thirteen genes exhibited no change in expression levels when light treatment for 30 min, but with changed expression level as increasing of light treatment (Type 3–7). After light treatment for 30 min, six genes showed an initial increase, followed by the stabilized expression pattern (Type 3), while the expression level of three genes (Type 4) increased firstly and then returned to the starting level. In Type 6, two genes exhibited an initial decrease, and then returned to the starting level. Type 5 and Type 7 each contained one gene which was up-regulated or down-regulated after light treatment for four hours.

**Figure 7:**
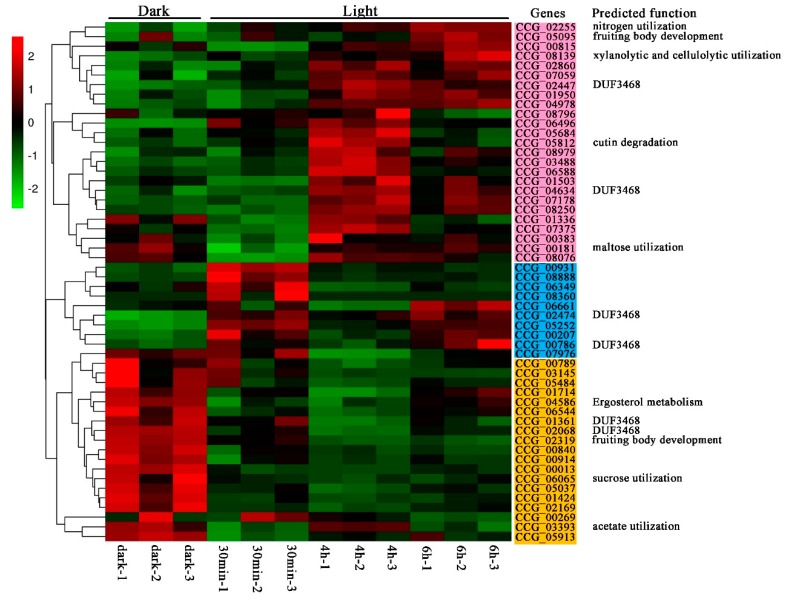
Expression levels of C6 zinc domain genes under different light conditions in *T. guangdongense*. The heatmap was constructed based on the FPKM values of C6 zinc domain genes. Genes immediately up-regulated by light are represented in pink, and immediately down-regulated by light are represented in yellow. Genes up-regulated by light after treatment for more than four hours are represented in blue.

Based on the RNA-seq data, a heatmap of 54 differentially expressed C6 zinc genes, represented by FPKM (fragments per kilobase of transcript per million mapped reads) values under different light conditions, was established (Figure 7). In comparison with the dark condition, 64.8% of the total differentially expressed genes were up-regulated by light, while the rest of the genes were down-regulated. Of the up-regulated genes, 46.3% of genes represented by pink were up-regulated after light treatment for more than 30 min, while 18.5% of genes represented by blue were up-regulated immediately after exposure to light. Among the up-regulated genes, four genes (CCG_02255, CCG_08139, CCG_05812, and CCG_00181) were predicted to be involved in metabolic processes, including nitrogen utilization, xylanolytic and cellulolytic utilization, cutin degradation, and maltose utilization. Of the down-regulated genes, three genes (CCG_08139, CCG_05812, and CCG_00181) were predicted to be involved in metabolic processes, including ergosterol biosynthesis or uptake, sucrose utilization, and acetate utilization. Among genes predicted to be involved in the asexual/sexual developmental process, CCG_05095, which was highly homologous to *POR1*, was up-regulated after exposure to light for more than 30 min; while CCG_02319, which was highly homologous to *OEFC1*, was down-regulated after light treatment. 

## 4. Discussion

Untill now, C6 zinc genes have only been identified in fungi and yeast [1,2,51,52], and belong predominantly to the ascomycete family, as only one has been characterized in the basidiomycete family [53]. In *S. cerevisiae*, 55 members of this family have been identified [1,54], while in the human fungal pathogen *C. albicans*, genome sequence analysis predicted that 77 putative C6 zinc genes existed in this species [55]. C6 zinc genes are also found in other fungi [1,56], but only a few of them have been studied in detail. Chang and Ehrlich [4] provided an overview of C6 zinc genes in *A. flavus*, with up to 306 members estimated by automatic and manual analyses. In *Cordyceps* as well, C6 zinc genes have been partially predicted according to the genome sequence of *C. militaris*, and the expression levels of some C6 zinc genes were shown to be affected by light [57,58]. In view of the limited available evidence, more research is needed to further identify the C6 zinc genes and evaluate their functions. This work described the C6 zinc cluster gene family in *T. guangdongense*, and compared the gene numbers, C6 variable regions, and type of protein domains with other two allied species (*C. militaris* and *O. sinensis*). Among the three species, *C. militaris* had the maximum number of C6 zinc domain genes, followed by *T. guangdongense*, while *O. sinensis* had the minimum number of C6 zinc domain genes possibly due to the relatively incomplete genome sequence. However, comparison to the other fungi, the maximum number of C6 zinc genes in *Cordyceps militaris* (169) was less than that in *A. flavus*, but more than those in *C. albicans* and in *S. cerevisiae*. These results provided vital information to prompt the functional investigations and mechanism exploration of C6 zinc proteins.

C6 zinc genes could be classified into several subgroups based on the variable region of CysX_2_CysX_6_CysX_5-16_CysX_2_CysX_6-8_Cys. The C6 zinc proteins shared a similar distribution pattern of C6 variable regions, with C-2-C-6-C-6-C-2-C-6-C being the most abundant pattern among *T. guangdongense*, *C. militaris* and *O. sinensis*, followed by C-2-C-6-C-5-C-2-C-6-C, C-2-C-6-C-5-C-2-C-8-C and C-2-C-6-C-8-C-2-C-6-C. This phenomenon is also similar to those in *Aspergillus* species [4], indicating that the structures of the variable C6 proteins among ascomycete fungi are evolutionarily conserved. There are some exceptions to this rule, for example, patterns with the number of amino acid residues in C6 variable regions between C3 and C4 exceeding twelve were not discovered in *C. militaris*, but were presented in *T. guangdongense*. While other patterns in *C. militaris*, like C-2-C-6-C-8-C-2-C-7-C and C-2-C-6-C-5-C-2-C-12-C, were not found in *T. guangdongense*. It could be due to the incomplete genome sequence or due to subtle differences among different species. 

As mentioned earlier, most C6 zinc genes were shown to participate in regulating multiple primary metabolic processes, such as carbon utilization, nitrogen utilization, amino acid metabolism, gluconeogenesis and respiration in yeast, as well as in other fungi, like *A. nidulans*, *A. oryzae*, *A. niger*, *N. crassa*, *Trichoderma reesei*, *Nectria hematococca*, *Kluyveromyces lactis*, *Hansenula polymorpha*, and *C. albicans* [1]. Besides, a minority of C6 zinc genes were related to the secondary metabolism production, such as *AFLR*, *FUM21*, *GLIZ*, *CTB8*, *LOVE*, *APDR*, *AFOA*, and *MdpE* so on [4]. In this work, we predicted that 29 C6 zinc genes were involved in primary metabolic processes, and also found two C6 zinc genes (CCG_07856 and CCG_07862) predicted to be located at the Indole-T1pks type gene cluster, which encoded proteins were highly homologous to the proteins involved in viridicatumtoxin biosynthesis [50]. However, it is unclear if both or only one of the two transcription factors are involved in the metabolic regulatory process of the corresponding cluster. Furthermore, according to the previous studies, many of these C6 zinc genes may also have overlapping functions, and sometimes they may work together to regulate a different metabolic process. Therefore, further confirmed the functions of these C6 zinc genes will be a challenge.

Analysis of the protein secondary structure indicated that C6 zinc proteins could be categorized into three groups. Of note, among the identified C6 zinc cluster genes, few encode a unique domain called DUF3468. As mentioned earlier, the DUF3468 domain is a transcriptional activation domain, and DUF3468 proteins are involved in asexual conidiation and sexual differentiation of *A. nidulans* and *A. flavus* [4]. In this study, five genes (CCG_05095, CCG_04350, CCG_01961, CCG_02319, and CCG_01824), which encoding proteins contained a DUF3468 domain, were predicted to be associated with asexual and sexual developmental processes, especially for the *PRO1* (encodes a C6 zinc finger protein with a typical DNA binding domain of Gal4-like C6 zinc finger proteins) homologous gene CCG_05095. The amino acid sequence of CCG_05095 was highly homologous to the PRO1 proteins in other fungi. *PRO1* exists in most ascomycetes in the form of a single gene [59], but two *PRO1* homologs are present in *A. nidulans* that act as either repressor (*RosA*) [37] or inducer (*NosA*) of sexual development [60]. In this work, only one *PRO1* homologous gene was identified in *T. guangdongense*, however, the other two genes CCG_04350 and CCG_01824 were closely related to *PRO1A* in *N. crassa* [18]. PRO1 was firstly identified as a TF that controls the developmental switch from young to mature fruiting bodies in the filamentous fungus *S. macrospora* [61]. Steffens et al. [59] further investigated the genome-wide regulatory network controlled by *PRO1* by employing chromatin immunoprecipitation combined with next-generation sequencing (ChIP-seq), and found that a large number of genes, involved in sexual development in *S. macrospora* and other filamentous ascomycetes, were *PRO1* targets genes. From the fact that *PRO1* acted as a master regulator of genes for signaling components that controlled fruiting body formation in fungi [59], we think that the *PRO1* homologous gene CCG_05095 was mainly involved in the sexual development, however, its function as repressor or inducer of sexual development needs to be confirmed. 

Light is a pervasive environmental signal serving either as a source of energy, or information for the adaptation of biological processes to essentially all branches of life [62,63,64]. Fungal species have been shown to respond to light, ranging from developmental decision making to metabolic reprogramming to pathogenesis [65,66,67,68]. Fungi can sense near-ultraviolet, blue, green, red and far-red light using up to 11 photoreceptors to control a large proportion of the genome and thereby adapt to environmental conditions [64]. In macrofungi, light is a vital factor, especially for edible mushrooms, and it is required for the formation of the fruiting body primordium and the development of fruiting body. In *C. militaris*, the essential role of light in fruiting body development and certain metabolite production was demonstrated, and C6 type transcription factors were the main downstream regulators involved in the fungal light reaction [58]. The C6 type transcription factor CCM_02196, a homologous gene of *PRO1*, was light-regulated, and acted as target of blue-light receptor gene *CmWC-1*, as well as other C6-type TFs (CCM_01467, CCM_04014, CCM_02196, CCM_07587, CCM_04849, and CCM_05610) [58]. Hence, we investigated the expression of C6-type genes under different light conditions. We found that 54 C6-type genes were light-regulated in *T. guangdongense*, and the expression levels of some genes were significantly different after light exposure for 30 min, indicating upstream regulatory genes for photoreceptors, while expression levels of others were significantly different till light exposure for four hours, implying downstream regulatory genes for photoreceptors. In these light-regulated genes, two of them (CCG_02319 and CCG_05095) were predicted to be related to fruiting body development, and ten (CCG_00181, CCG_02255, CCG_03393, CCG_04586, CCG_05812, CCG_06056, CCG_08139) were predicted to be metabolism associated genes. Our expression analyses suggest that light influences fruiting body development in *T. guangdongense*, and may also affect several other metabolic processes, including carbon, nitrogen and acetate utilization, and ergosterol biosynthesis and uptake, by activation or suppression of related C6 zinc genes. Nevertheless, more detailed information and functional annotation of other differentially expressed genes are urgently needed.

## Figures and Tables

**Table 1:** Classification and comparison of C6 Zinc proteins based on the Zinc cluster DNA-binding domains in three species.

Subgroup	*Tolypocladium guangdongense*	*Cordyceps ilitaris*	*Ophiocordyceps sinensis*
C-2-C-6-C-6-C-2-C-6-C	65	83	24
C-2-C-6-C-5-C-2-C-6-C	26	26	4
C-2-C-6-C-5-C-2-C-8-C	15	10	7
C-2-C-6-C-8-C-2-C-6-C	11	14	3
C-2-C-6-C-9-C-2-C-6-C	6	8	1
C-2-C-6-C-7-C-2-C-6-C	5	7	0
C-2-C-6-C-12-C-2-C-6-C	2	2	0
C-2-C-6-C-5-C-2-C-7-C	2	2	0
C-2-C-6-C-10-C-2-C-12-C	1	0	1
C-2-C-6-C-10-C-2-C-6-C	1	2	2
C-2-C-6-C-14-C-2-C-6-C	1	0	0
C-2-C-6-C-17-C-2-C-6-C	1	0	0
C-2-C-6-C-5-C-2-C-11-C	1	4	2
C-2-C-6-C-5-C-2-C-5-C	1	0	0
C-2-C-6-C-5-C-2-C-9-C	1	1	0
C-2-C-6-C-6-C-2-C-7-C	1	1	1
C-2-C-6-C-6-C-2-C-8-C	1	5	0
C-2-C-6-C-9-C-2-C-7-C	1	1	0
C-2-C-6-C-11-C-2-C-7-C	0	1	0
C-2-C-6-C-12-C-2-C-6-C	0	1	0
C-2-C-6-C-6-C-2-C-7-C	0	1	0
C-2-C-6-C-8-C-2-C-8-C	0	7	0
C-2-C-6-C-5-C-2-C-12-C	0	2	1
C-2-C-6-C-6-C-2-C-11-C	0	1	0
C-2-C-6-C-6-C-2-C-9-C	0	1	0

**Table 2:** Homologous analysis of C6 zinc proteins with the metabolic process in *T. guangdongense*.

Gene_ID	Blast Result	Putative Gene Product Function	Role	References
CCG_08139	*xlnR*	Controls expression of xylanolytic enzymes genes	xylanolytic and cellulolytic utilization	[37]
CCG_06056	*SUC*1	Regulates sucrose metabolic genes	sucrose utilization	[38]
CCG_06421	*SUC*1	Regulates sucrose metabolic genes	sucrose utilization	[38]
CCG_02169	*SUC*1	Regulates sucrose metabolic genes	sucrose utilization	[38]
CCG_00181	*-*	maltose O-acetyltransferase	maltose utilization	/
CCG_08276	*-*	chitinase 1 precursor	Chitin degradation	/
CCG_05812	*ctf1β*	Activator of cutinase genes	Cutin degradation	[39]
CCG_01013	*UPC2*	activator of ergosterol biosynthetic genes	Ergosterol metabolism	[40]
CCG_04586	*UPC2*	activator of ergosterol biosynthetic genes	Ergosterol metabolism	[40]
CCG_01961	*ECM22*	Activator of ergosterol biosynthetic genes	Ergosterol metabolism	[12]
CCG_02464	*nit-4*	Activator of the nitrate assimilatory pathway	nitrate assimilation	[41]
CCG_08116	*nit-4*	Activator of the nitrate assimilatory pathway	nitrate assimilation	[41]
CCG_09094	*nirA*	Regulator of nitrate assimilation	nitrate assimilation	[42]
CCG_02875	*OTam/TamA*	Involved in nitrogen regulation	nitrogen utilization	[43]
CCG_02255	*DAL81*	Activator of nitrogen catabolic genes	nitrogen utilization	[44]
CCG_04992	*acu-15*	Transcriptional activator protein	acetate utilization	[45]
CCG_00410	*acu-15*	Transcriptional activator protein	acetate utilization	[45]
CCG_03393	*acu-15*	Transcriptional activator protein	acetate utilization	[45]
CCG_04678	*acu-15/FacB*	Activator of acetate regulatory genes	acetate utilization	[45]
CCG_04262	*LEU3*	Activator/repressor of leucine biosynthesis genes	Amino acid metabolism	[46]
CCG_06591	*MET32*	Transcriptional regulator involved in sulfate assimilation and sulfonate metabolism	sulfate assimilation and sulfonate metabolism	[47]
CCG_02459	*-*	Positive regulator of purine utilization	purine utilization	/
CCG_04993	*-*	Pyrimidine pathway regulatory protein 1	Pyrimidine utilization	/
CCG_02072	*-*	Quinic acid utilization activator	Quinic acid utilization	/
CCG_00730	*Trz1*	tRNA processing endoribonuclease Trz1	RNase Z activity	[48]
CCG_05829	*fcf1*	rRNA-processing protein fcf1	pre-rRNA processing	[49]
CCG_06615	*-*	stress gene activator	stress response	[29]
CCG_07862	*vrtR1*	Viridicatumtoxin synthesis protein R1	viridicatumtoxin biosynthesis	[50]
CCG_07856	*vrtR2*	Viridicatumtoxin synthesis protein R2	viridicatumtoxin biosynthesis	[50]

## Supplementary Materials

The following are available online at https://www.mdpi.com/2073-4425/10/3/179/s1, Table S1: Statistics of fungal specific transcription factors, C6-protiens and C6-TFs in *T. guangdongense* (CCG), *C. militaris* (CCM) and *O. sisensis* (OCS). Table S2: Detail information of fungal specific transcription factors (FSP), C6-protiens and C6-TFs in *T. guangdongense* (CCG), *C. militaris* (CCM) and *O. sisensis* (OCS). Table S3: Variable subregion of the identified C6-TFs gene family members in *T. guangdongense* (CCG), *C. militaris* (CCM) and *O. sisensis* (OCS). Table S4: Characteristics of the identified C6-TFs gene family members in *T. guangdongense*. Table S5: Blast analysis of the identified C6 zinc proteins in *T. guangdongense*. Table S6: List of the identified mofit of DUF3468 domain proteins in *T. guangdongense*. Table S7: FPKM values of the differential expression C6 zinc cluster-encoding genes in *T. guangdongense* produced by RNA-seq. Figure S1 Alignment of amino acid sequences of *T. guangdongense* PRO1 (TgPRO1) and its orthologs in *N. crassa*, *S. macrospora*, *C. militaris*, and *A. nidulans*. The asterisk represents the cysteine in the C6 domain, and the nuclear localization signal (NLS) sequences are indicated by red line. Figure S2. The motif sequences in C6 proteins, which were identified by MEME.
