# Supplementary figures and images for: Genome-Wide Analysis of the Zn(II)2Cys6 Zinc Cluster-Encoding Gene Family in Tolypocladium guangdongense and Its Light-Induced Expression
Source: Genes (Basel). 2019 Feb 26;10(3):179. doi: 10.3390/genes10030179 (PMC6471507; doi:10.3390/genes10030179)

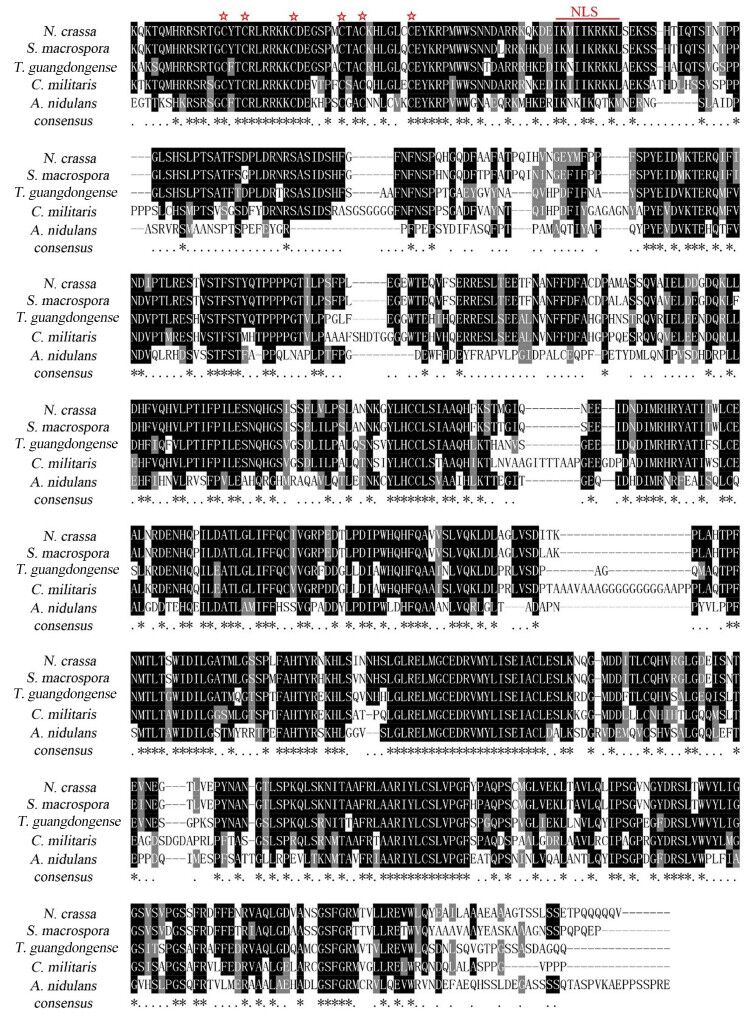

Supplement: Supplementary file 1 [file genes-10-00179-s001.zip › genes-435191-supplementary/genes-Supplementary file/Figure S1.jpg]

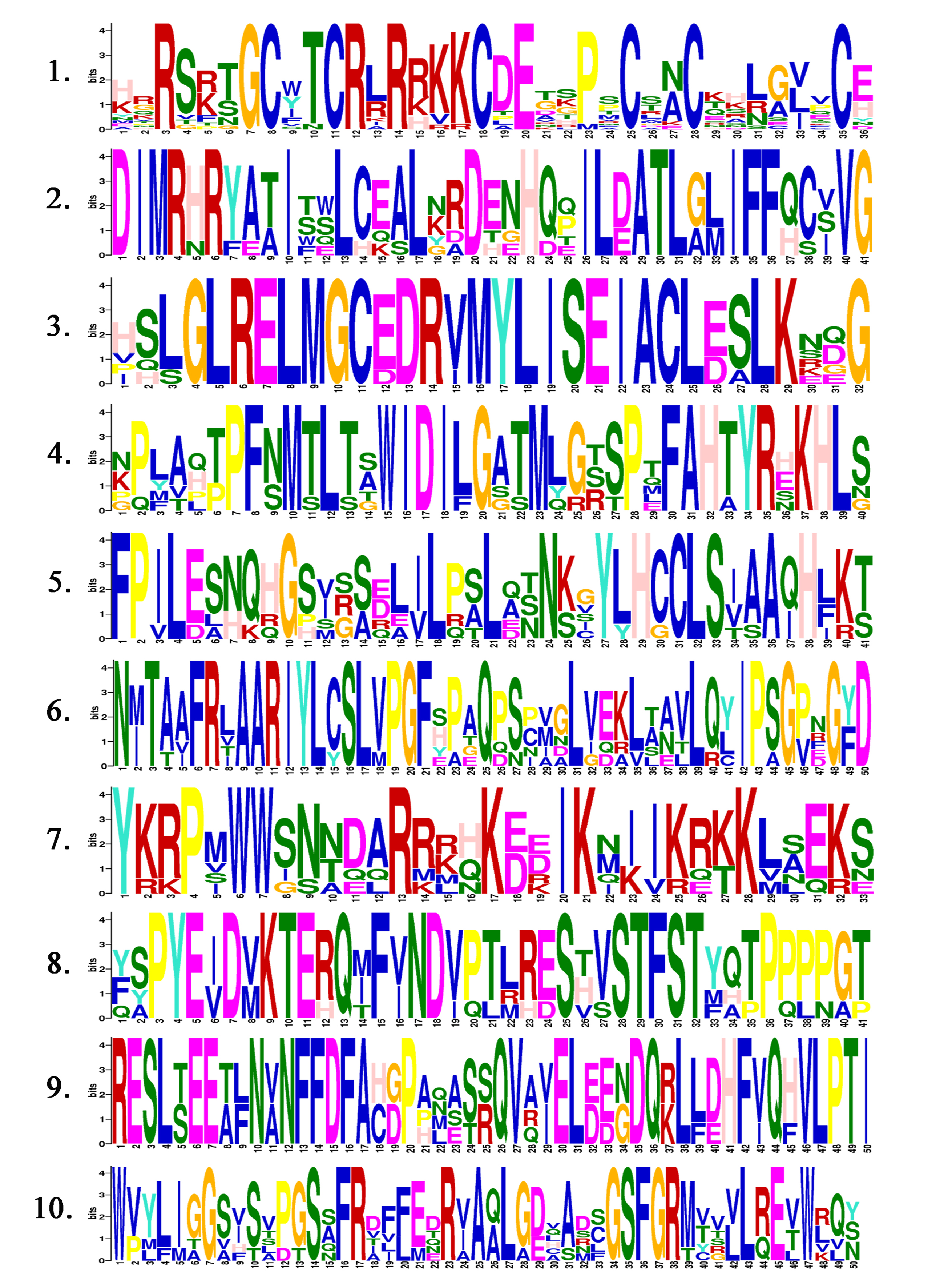

Supplement: Supplementary file 1 [file genes-10-00179-s001.zip › genes-435191-supplementary/genes-Supplementary file/Figure S2.jpg]
